# Risk factors associated with nocebo effects: A review of reviews

**DOI:** 10.1016/j.bbih.2024.100800

**Published:** 2024-05-22

**Authors:** Francesca Grosso, Diletta Barbiani, Cesare Cavalera, Eleonora Volpato, Francesco Pagnini

**Affiliations:** a Department of Psychology, Università Cattolica del Sacro Cuore, Milan, Italy; b IRCCS Fondazione Don Carlo Gnocchi, Milan, Italy

**Keywords:** Nocebo, Nocebo risk factors, Nocebo mechanisms, Meta-review

## Abstract

**Objective:**

This meta-review aims to identify and categorize the risk factors that are associated with nocebo effects. The nocebo effect can exert a negative impact on treatment outcomes and have detrimental outcomes on health. Learning more about its potential predictors and risk factors is a crucial step to mitigating it.

**Methods:**

Literature review studies about the risk factors for nocebo effects were searched through five databases (PubMed, Scopus, The Cochrane Library, PsycINFO, and Embase) and through grey literature. Methodological validity and risk of bias were assessed. We conducted a thematic analysis of the results of the forty-three included reviews.

**Results:**

We identified nine categories of risk factors: prior expectations and learning; socio-demographic characteristics; personality and individual differences; neurodegenerative conditions; inflammatory conditions; communication of information and patient-physician relationship; drug characteristics; setting; and self-awareness. We also highlighted the main biochemical and neurophysiological mechanisms underlying nocebo effects.

**Conclusions:**

Nocebo effects arise from expectations of adverse symptoms, particularly when triggered by previous negative experiences. A trusting relationship with the treating physician and clear, tailored treatment instructions can act as protective factors against a nocebo effect. Clinical implications are discussed.

## 1 Introduction

The nocebo effect is a phenomenon in which an individual experiences negative side effects from a treatment or procedure, even though it contains no medically active ingredients. This negative outcome is a result of the person's negative expectations and beliefs about the treatment, rather than any actual physical property of the treatment itself (Evers et al., 2018, Evers et al., 2018b). The term “nocebo effect” was originally coined to indicate the negative counterpart of the placebo effect and to distinguish the adverse from the beneficial effects of placebos (Faasse et al., 2013): treatment-related nonspecific factors can elicit placebo effects when they have positive meaning and nocebo effects when they hold a negative connotation, leading in this latter case to a worsening of symptoms. For example, negative expectations can induce an increased risk of developing various health-related conditions, including respiratory diseases (Vlemincx et al., 2021), pain (Manaï et al., 2019), gastrointestinal symptoms (Ma et al., 2019a, Ma et al., 2019b), influenza-like symptoms (Pagnini, 2019), and postoperative morbidity (Maroli et al., 2022).

Although not always distinct, researchers examine two variants of nocebo effects: primary nocebo effects and nocebo side effects (Faasse et al., 2013). Primary nocebo effects refer to the effects as the primary negative outcome of a treatment/medical procedure intended as harmful. Such outcomes were described by Hahn as nocebo effects (Hahn, 1997), which were distinguished from ‘placebo side effects’, whereby a treatment primarily intended as beneficial can cause harmful outcomes. This is the case of nocebo side effects, namely, unpleasant symptoms that arise following a treatment that is primarily intended as beneficial, but of which specific side effects are anticipated (Meeuwis et al., 2021). Notably, recent evidence suggests that primary manipulations of nocebo and nocebo side effects do not produce equivalent results. Caplandies et al. (2017) demonstrated how instructions on the nocebo effect can produce different outcomes depending on whether the adverse effect is described as a primary effect or as a treatment side effect (Faasse et al., 2013). Nocebo effects are prompted in research but, unlike placebo effects, are not purposefully elicited in clinical practice, since this would undermine the basic ethical standards of beneficence and non-maleficence.

The phenomenon of nocebo effects, characterized by the adverse outcomes resulting from negative expectations and beliefs, has garnered increasing attention in recent years (Colloca and Benedetti, 2016). The potential impact of nocebo effects on treatment outcomes necessitates the development of protocols to effectively communicate the risks associated with specific treatments, thus minimizing the occurrence of these effects. Considering its implications for clinical practice, patient well-being, and healthcare costs, understanding the underlying mechanisms of the nocebo phenomenon is of paramount importance (Rodríguez-Monguió et al., 2003; Webster et al., 2016). The pervasiveness of the nocebo phenomenon and its potential consequences highlight the need to further elucidate the key factors that predispose, sustain, or exacerbate it. It is crucial to stay abreast of the rapidly evolving literature in this field (Weimer et al., 2022) and continually update our understanding to incorporate the latest findings.

The interest on nocebo effects has greatly increased over time - for example, a PubMed search through keywords provided 1 article in 1961, which increased to 17 in 2007, to reach 154 in 2022. Considering this context, the present paper aims to provide an up-to-date review of reviews published within the last 12 years. By summarizing and systematizing the newest findings from these reviews, we seek to identify and consolidate the primary factors that predict or act as catalysts for nocebo effects. These include psychosocial and clinical risk factors, as well as neurobiological moderators and mediators that can represent the underlying mechanisms.

## 2 Materials and methods

### 2.1 Protocol registration and eligibility criteria

Our search strategy followed the Preferred Reporting Items for Systematic Reviews and Meta-Analysis (Page et al., 2021). The protocol was registered on the International Prospective Register of Systematic Reviews. Only literature reviews (systematic review, meta-analysis, scoping review, and mini review) investigating nocebo effects and published from 2012 onwards were considered. We excluded unavailable full texts, conference proceedings, abstracts, commentaries, editorials, opinions, book chapters, journal articles, and debates. Literature was limited to studies in the English language involving humans, including adults over 18 years old.

### 2.2 Search methods for identification of reviews

The research included reviews published from 2012 to April 2024, in which nocebo risk factors were considered. We conducted a systematic search on PubMed (National Library of Medicine and National Institutes of Health), Scopus, The Cochrane Library, PsycINFO, and Embase. Grey literature documents, identified via Google Scholar and OSFHome, ArXiv, SocArXiv, PsycArXiv, and MedArXiv databases, were also included. Keywords and text words used in the search for each of the considered databases were “nocebo effects*” OR “nocebo mechanism*” combined with “risk factors” OR “mediators” OR “moderators”.

### 2.3 Review selection

Two authors (F.G. and C.C.) independently screened the title, abstract, and key terms in the first instance for potential inclusion followed by a full-text screening and data extraction. Data extraction included the following procedures: Level 1 - Title and abstract screening: title and abstracts identified by the electronic database searches were screened for potential inclusion; in cases where a decision for exclusion or potential inclusion could not be made by the title/abstract, the full text was retrieved; Level 2 - Full-text screening: full-text articles of the included reviews were then retrieved and further screened based on inclusion and exclusion criteria; disagreements were resolved by discussion at each stage in the process; if an agreement could not be reached, a third reviewer (F.P.) was consulted. In addition, reference list searches of included reviews were manually undertaken and screened as per the same selection process to identify further studies of relevance for inclusion.

### 2.4 Data extraction and management

Data from the selected reviews were inserted into an Excel template by two independent researchers (F.G. and C.C.). The following data were extracted and included in the template: bibliographic information of papers (i.e., authors, country, and year of publication), aim, and the results reported (risk factors and outcome). Any disagreement was discussed with a third author to reach an agreement. Article authors were reached via e-mail in case of missing information.

### 2.5 Assessment of risk of bias in included reviews

All included reviews were quality appraised by two reviewers (F.G. and C.C.) independently. Discrepancies were resolved through dialogue and consultation with a further reviewer (F.P.) when necessary. Critical appraisal involves considering the risk of potential for selection bias, information bias, measurement bias, or confounding. Full-text articles selected for data extraction were assessed for methodological validity and risk of bias using the Risk of Bias Assessment Tool for systematic review and meta-analyses developed by the National Heart, Lung, and Blood Institute (Mangano, 2004).

### 2.6 Strategy for data synthesis

**Fig. 1:**
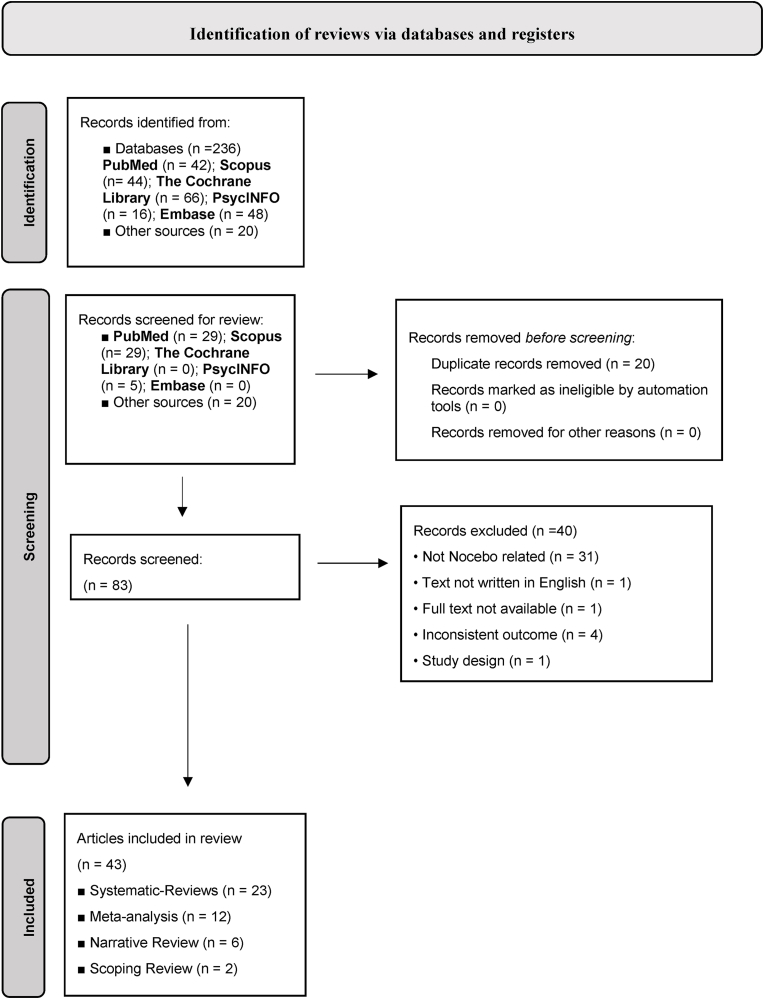
PRISMA Flow diagram.

We used thematic synthesis to summarize the results (Barnett-Page and Thomas, 2009). A reviewer (F.G.) coded the results of the included reviews and recorded the concepts that stood out as risk factors. All included reviews were re-read to ensure that relevant data were captured and integrated appropriately into the preliminary themes and sub-themes. All authors reviewed the preliminary analysis to ensure that key data had been captured from the included reviews and discussed the concepts to identify similarities and differences. The bridging of concepts across reviews was performed by grouping similar concepts and creating new ones if necessary. Firstly, ‘descriptive’ macro-themes specific to each review were identified, and as a second step, a thematic analysis based on an integrative approach was conducted to conceptually combine the most frequent descriptive themes that recurred across the individual reviews. In this latter process, the reviewers' effort was to go beyond the meaning of the descriptive themes to generate new explanations and interpretative hypotheses that converged into broader categories summarizing the nocebo risk factors. Separate files were created (F.G.) for each identified category, together with the citations of the reviews that emphasized the category in question. Emerging themes were then identified, and then sub-themes, to construct the 'core category'. The coding scheme required a circular approach, re-reading the articles several times and integrating new information that was initially left out, to also examine the relationships between the themes (F.G. and C.C.). In this way, it was possible to create links between and within the categories. We adopted a weight-of-evidence approach (Regoli et al., 2019), whereby the strength of evidence for each risk factor was identified based on the number of studies investigating it. Our search strategy resulted in the identification of forty-three reviews, for each of which we summarized and synthesized the risk factors related to the nocebo effects. The selection process is reported in Fig. 1.

## 3 Results

### 3.1 Included reviews

We identified 43 eligible reviews. Among the articles included, 23 were systematic reviews, 12 were meta-analyses, 6 were narrative reviews, and 2 were scoping reviews. Each review, along with its respective aims, results, and considered risk factors, is summarized in Table 1.

**Table 1:** Summary table.

#	REVIEW	AIMS	SAMPLE SIZES	OUTCOME	RISK FACTORS
1.	Manchikanti et al. (2011)	To explain placebo and nocebo in interventional pain management.	N = 120	The phenomena of placebo analgesia and nocebo hyperalgesia are not merely simple effect biases, but rather, they arise from neurophysiological processes that modulate the integration of nociceptive signals throughout the central nervous system.	■ Prior Expectations and Learning
2.	Data-Franco, J., & Berk, M. (2013)	To provide an overview of the nocebo effect, focusing on the recognition of its phenomenology, at-risk demographic profiles, clinical situations, and personality factors, as well as discriminating somatic symptoms in the general population from treatment-related adverse effects.		People who exhibit characteristics like neuroticism, pessimism, or type A personalities could be more susceptible to the nocebo effect. To address this occurrence in clinical settings, it is essential to recognize and comprehend it. This can be accomplished by adjusting the method by which potential negative effects of drugs are communicated, setting realistic patient expectations, and improving the relationship between patients and healthcare providers. By doing so, the nocebo effect can be managed more effectively.	■ Prior Expectations and Learning ■ Socio-demographic characteristics ■ Personality and individual differences ■ Communication of information and patient-physician relationship
3.	Feys et al. (2014)	To determine if unblinding in randomized controlled trials (RCTs) leads to enhanced placebo effects in the intervention groups and nocebo effects in the placebo groups. Additionally, the study aims to identify potential factors that may moderate these effects.	N = 23 887	In recent randomized clinical trials, a tendency has been observed that leads to an underestimation of the placebo effect, consequently generating a nocebo effect. However, there is no indication of an overestimation of the efficacy of interventions, which could potentially amplify placebo effects.	■ Prior Expectations and Learning
4.	Vase et al. (2014)	To fully understand the mechanisms underlying placebo and nocebo effects in chronic pain patients, it may be relevant to directly study the contribution of specific cognitions and feelings to the pain-relieving and pain-increasing effects.	Not Applicable (NA)	Expectations have a notable influence in forecasting the placebo and nocebo outcomes for symptoms like dyspnea and itch. However, their impact on fatigue and nausea is not entirely evident. In addition, personal traits do not uniformly forecast the placebo or nocebo effects in various symptoms or research.	■ Prior Expectations and Learning ■ Communication of information and patient-physician relationship
5.	Frisaldi, E., Piedimonte, A., & Benedetti, F. (2015)	To explore the psychological mechanisms and neurochemical networks involved in placebo and nocebo effects across different conditions, such as pain and motor disorders.	Not Applicable (NA)	The patient's psychological and social environment during treatment, which includes the therapeutic process ritual, can influence the patient's brain chemistry and neural pathways. Furthermore, the mechanisms that placebos and nocebos activate are similar to those triggered by medications, suggesting that cognitive and emotional factors can interfere with drug efficacy.	■ Setting ■ Communication of information and patient-physician relationship
6.	Symon, A., Williams, B., Adelasoye, Q. A., & Cheyne, H. (2015)	To discuss the existence, prevalence, and characteristics of the nocebo effect in healthcare.		The nocebo effect is found to be more prevalent in women and in situations where prior negative knowledge or expectations exist. Furthermore, pre-existing psychological traits such as anxiety, neurosis, panic disorder, or pessimism can exacerbate the effect.	■ Socio-demographic characteristics ■ Personality and individual differences ■ Prior Expectations and Learning
7.	Bartels, D.J.P., van Laarhoven, A.I.M., van de Kerkhof, P.C.M. and Evers, A.W.M. (2016)	To evaluate the current evidence for the placebo and nocebo effects on itch, and to investigate the potential similarities in the underlying mechanisms of these effects as compared to placebo and nocebo effects on pain.	Not Applicable (NA)	The combination of verbal suggestion and conditioning has proven to be more effective in inducing both placebo and nocebo effects as compared to suggestion alone.	■ Prior Expectations and Learning ■ Personality and individual differences
8.	Colloca, L. & Benedetti, F. (2016)	To understand the neuropeptides involved in nocebo effects Placebo and nocebo effects have emerged as one of the most interesting and elegant models to explore some of the descending endogenous modulatory systems	Not Applicable (NA)	Placebo and nocebo effects have emerged as one of the most interesting and elegant models to explore some of the descending endogenous modulatory systems	■ Prior Expectations and Learning ■ Self-awareness
9.	Planès et al. (2016)	To provide a comprehensive overview of the current state of knowledge on the nocebo effect.		The outcome of a particular treatment can be negatively impacted by nocebo effects, which are like how placebo effects can positively impact outcomes. Physicians' verbal and nonverbal communication often includes unintended negative suggestions that could potentially trigger nocebo effects.	■ Prior Expectations and Learning ■ Socio-demographic characteristics ■ Communication of information and patient-physician relationship ■ Drug characteristics
10.	Webster, R. K., Weinman, J., & Rubin, G. J. (2016)	To identify the risk factors involved in the development of nocebo effects.		The most significant determinants of nocebo effects were a perception of receiving a higher dose of exposure, receiving explicit suggestions that the exposure triggers symptoms or arousal, witnessing other individuals experiencing symptoms due to the exposure, and holding higher expectations of experiencing symptoms.	■ Prior Expectations and Learning ■ Neurodegenerative conditions ■ Self-awareness
11.	Blasini, M., Corsi, N., Klinger, R., & Colloca, L. (2017)	To conduct a comprehensive review of the published literature on analgesia and hyperalgesia caused by negative expectations and the nocebo effect.	Not Applicable (NA)	The experience of adverse effects resulting from negative expectations about a treatment or medication is known as the nocebo effect. This phenomenon is influenced by a variety of factors, including verbal suggestions, prior experiences, and observation of pain. Other factors that can contribute to the nocebo effect include genetic factors, learning processes, personality traits, psychological factors, and environmental cues. Negative expectations can be shaped by the behavior of medical providers and the use of medical devices, both of which can influence the perception of pain.	■ Prior Expectations and Learning ■ Personality and psychological differences ■ Communication of information ■ Setting
12.	Dodd, S., Dean, O. M., Vian, J., & Berk, M. (2017)	To investigate the current knowledge regarding the theoretical and biological underpinning of the nocebo and placebo phenomena.	Not Applicable (NA)	Conditioning, expectancy, and personal traits have a substantial influence on the magnitude of their impact and may differ depending on the unique features of the experimental design and individual differences. Furthermore, the neurobiological mechanisms that give rise to the nocebo and placebo phenomena are also examined.	■ Prior Expectations and Learning ■ Personality and individual differences ■ Communication of information and patient-physician relationship ■ Setting
13.	Vambheim S.M. & Magne F. (2017)	To investigate whether there are systematic sex differences in the placebo and the nocebo effect.	N = 1264	This review suggests that there are gender disparities in placebo and nocebo effects, which are likely due to variations in stress, anxiety, and the endogenous opioid system.	■ Socio-demographic characteristics ■ Personality and individual differences
14.	Koban et al. (2017)	To discuss and integrate prominent examples including observational learning, social influence, and brain mechanisms that have been studied in partially separate literature.	Not Applicable (NA)	The impact of instructions and social information on an individual's emotional experience may be mediated by changes in their expectation and appraisal, which involve assessing the personal significance and effect on their well-being. As per a proposed model, the dorsolateral and ventromedial prefrontal cortex regions in the prefrontal area may regulate emotional processing based on instructions and expectations transmitted through social means.	■ Prio Expectations and Learning ■ Personality and individual differences ■ Setting
15.	M. Silva et al. (2017)	To assess the placebo and nocebo responses in restless legs syndrome (RLS) and investigate the factors that influence them.	N = 5046	The placebo response in restless legs syndrome (RLS) surpasses the threshold of minimal clinically important difference, and the occurrence of adverse events is notable as well.	■ Neurodegenerative conditions ■ Inflammatory conditions
16.	Howick and Hoffmann (2018)	To examine the prevalence of adverse events in placebo groups in trials across various conditions.	N = 250, 726	Adverse events can be caused by negative expectations. Clinicians can minimize nocebo effects in clinical trials by being mindful of symptom suggestions. Informed consent that respects patient autonomy and avoids causing harm should be tailored to meet individual needs.	■ Prior Expectations and Learning ■ Setting ■ Communication of information and patient-physician relationship
17.	Kravvariti et al. (2018)	To investigate the incidence of adverse events resulting in treatment withdrawal by placebo-arm participants in randomized controlled trials (RCTs) of patients with rheumatic and musculoskeletal diseases (RMDs), and to evaluate the potential contribution of nocebo effects in healthcare to these events.	Not Applicable (NA)	Intrinsic differences between drugs may result in a lack of response in some patients, while nocebos may also play a significant role in low biosimilar retention rates. Therefore, rheumatologists and allied healthcare professionals must be aware of and identify potential nocebo effects early on.	■ Socio-demographic characteristics ■ Personality and individual differences ■ Neurodegenerative conditions ■ Communication of information and patient-physician relationship ■ Drug characteristics ■ Setting
18.	Rossettini, G., Carlino, E., & Testa, M. (2018)	To explore the psycho-neurobiological mechanisms underlying the modulation of pain by placebo and nocebo effects, and to investigate the potential effects of these phenomena on neurobiological, perceptual, and cognitive processes in the body.	Not Applicable (NA)	Various explanatory models, including classical conditioning and expectancy, can elucidate how contextual factors (CFs) give rise to placebo and nocebo effects. These CFs operate via distinct neural networks and neurotransmitters that have been identified as mediators of both placebo and nocebo effects.	■ Prior Expectations and Learning ■ Setting
19.	P. Zis & D. (2020)	To investigate nocebo effects in non-traumatic brain diseases.		A widespread presence of the nocebo effect was revealed in diverse brain disorders, such as headaches, Parkinson's disease, Alzheimer's disease, depression, epilepsy, multiple sclerosis, and motor neuron disease.	■ Neurodegenerative conditions
20.	P. Zis et al. (2018)	To investigate the occurrence of adverse events (AEs) after the administration of placebos in randomized clinical trials designed to assess chronic inflammatory demyelinating polyneuropathy (CIDP).	N = 96	A notable proportion of placebo-treated patients reported experiencing at least one adverse event leading to a small percentage discontinuing placebo treatment due to AEs. Interestingly, all CIDP trial participants reported similar AEs, irrespective of their assigned study arm. In summary, the nocebo effect in CIDP appears to be significantly smaller when compared to other neurological diseases.	■ Neurodegenerative conditions
21.	Colloca, L., Panaccione, R., & Murphy, T. K. (2019).	To map the available literature on nocebo effects with biosimilars.		When patients are presented with biosimilars as potential treatment options, it is advisable to incorporate nocebo-reducing strategies to prevent negative expectations. Such strategies may include providing unbiased information on the risk-benefit profiles of the biosimilars, emphasizing their positive attributes, and promoting shared decision-making processes while empowering the patient.	■ Prior Expectations and Learning ■ Setting ■ Inflammatory conditions
22.	Daniali, H., & Flaten, M. A. (2019)	To investigate the effects of experimenter/clinician characteristics and nonverbal behavior on pain, placebo, and nocebo effects	N = 4275	The characteristics and nonverbal behaviors of clinicians or experimenters can influence the induction and alteration of pain, placebo, and nocebo effects.	■ Communication of information and patient-physician relationship ■ Drug characteristics ■ Setting
23.	Hansen, E., & Zech, N. (2019)	To examine the impact of language and physician-patient interaction on patient outcomes in medical situations.	Not Applicable (NA)	By enhancing their education and awareness of nocebo effects and negative patient expectations, healthcare providers can enhance their ability to identify and manage these phenomena.	■ Prior Expectations and Learning ■ Communication of information and patient-physician relationship ■ Drug characteristics ■ Setting
24.	Leal Rato et al. (2019)	To estimate the magnitude of the nocebo effect in Parkinson's disease and explore possible associations with study characteristics.	N = 8743	The increased likelihood of negative outcomes observed in patients who receive a placebo treatment implies that the current analysis of safety data may be distorted by the nocebo response.	■ Neurodegenerative conditions
25.	Manaï et al. (2019)	To highlight the underlying mechanisms and potential factors that contribute to a person's susceptibility to the nocebo effect about pain and associated outcomes, and to propose strategies that can prevent, reduce, or eliminate nocebo effects in clinical settings.	Not Applicable (NA)	Several recommendations and strategies have emerged from clinical and experimental evidence aimed at reducing the nocebo effect and improving pain management. These include furnishing patients with comprehensive information, improving communication and the patient-physician relationship, and offering psychoeducational support to patients to help them develop coping mechanisms for managing their expectations.	■ Socio-demographic characteristics ■ Personality and individual differences ■ Communication of information
26.	Petrie, K. J., & Rief, W. (2019)	This review discusses the impact of the placebo and nocebo effects on healthcare.	Not Applicable (NA)	Contextual factors and expectations are the fundamental drivers of placebo and nocebo effects as well as clinician-patient interaction	■ Prior Expectations and Learning ■ Setting ■ Communication of information and patient-physician relationship
27.	Spanou, I., Mavridis, T., & Mitsikostas, D. D. (2019)	To investigate the presence of the nocebo in generics and biosimilar substitution studies in some of the most common neurological diseases.		The full scope of the adverse consequences of the nocebo effect and placebo effect on patients with neurological conditions using generic and biosimilar medications is not yet fully understood based on current research. Moreover, there appears to be a knowledge gap between healthcare professionals and patients when it comes to the usage of generic and biosimilar drugs.	■ Neurodegenerative conditions ■ Drug characteristics ■ Inflammatory conditions
28.	Wolters, F., Peerdeman, K. J., & Evers, A. W. M. (2019)	To explore placebo and nocebo effects to four common symptoms: dyspnea, fatigue, nausea, and itching.		The impact of expectations on placebo and nocebo effects for dyspnea and itching is widely acknowledged, but it seems to have a weaker effect on fatigue and nausea. Additionally, there is no uniform pattern of personal characteristics that can predict placebo or nocebo effects in various symptoms or in diverse research studies.	■ Prior Expectations and Learning ■ Personality and individual differences
29.	C. Ma et al., 2019a, Ma et al., 2019b	To determine the combined occurrence rate of adverse events in patients assigned to placebo versus those receiving active therapy.	N = 16 978	Patients participating in randomized controlled trials for inflammatory bowel disease (IBD) frequently report adverse events (AEs) regardless of whether they are assigned to placebo or active treatment. Notably, there were no substantial differences observed in clinically significant AEs, serious adverse events, or withdrawals due to AEs between the two groups.	■ Neurodegenerative conditions ■ Inflammatory conditions
30.	Kern et al. (2020)	To provide a systematic review of the influence of personality traits on placebo and nocebo effects in controlled and uncontrolled studies.		There may be a correlation between optimism and the placebo effect. Studies have primarily focused on exploring the relationship between optimism and the Big Five personality traits, including neuroticism, extraversion, openness to experience, agreeableness, and conscientiousness. On the other hand, findings have suggested that higher anxiety levels are related to an increased risk of experiencing nocebo effects.	■ Prior Expectations and Learning ■ Personality and individual differences
31.	Smith, L. E., Webster, R. K., & Rubin, G. J. (2020)	To identify factors associated with side-effect expectations.	N = 16 549	The use of verbal risk descriptors (e.g., 'common') in comparison to numerical descriptors (e.g., percentages), along with a lower quality of life or well-being and ongoing symptoms, were found to be associated with elevated expectations of experiencing side effects.	■ Personality and individual differences ■ Communication of information and patient-physician relationship
32.	I. Skyt et al. (2020)	To explore the role of neurotransmitter systems in individuals undergoing experimental or acute postoperative pain, as well as in patients experiencing chronic pain.	N = 2284	The endogenous opioid system's role in placebo effects among healthy participants has been extensively studied, revealing clear positive indications of endogenous opioid release contributing to these effects. Some research suggests the involvement of the endocannabinoid and vasopressinergic systems, while findings for the dopaminergic and oxytocinergic systems are inconclusive.	■ Neurodegenerative conditions
33.	L. Colloca & A. Barsky. (2020)	To summarize and outline the implications of placebo and nocebo phenomena in neurobiological research, clinical practice, and the design and conduct of clinical trials.	Not Applicable (NA)	Patients receiving placebo in clinical trials discontinue it due to side effects, suggesting how a nocebo effect may contribute to discontinuation or lack of adherence to active treatments.	■ Prior Expectations and Learning ■ Neurodegenerative conditions ■ Inflammatory conditions
34.	Meeuwis et al. (2021)	To investigate how interindividual differences, expectations, placebo effects, and nocebo effects are interrelated to further understand the way interindividual differences may contribute to placebo and nocebo responding.	N = 112	In an open-label context, the nocebo effect on itch was influenced by expectations, but not in a closed-label context. These findings suggest that a lack of awareness of bodily sensations and the level of activation of the behavioral system may influence placebo and nocebo effects.	■ Prior Expectations and Learning ■ Personality and individual differences ■ Self-awareness
35.	F. D'Amico et al., 2021a, D'Amico et al., 2021b	To conduct a literature overview to summarize information on the nocebo effect in the inflammatory bowel disease (IBD) population.	N = 994	The efficacy and safety of biosimilars was demonstrated.	■ Inflammatory conditions
36.	Bagarić, B., Jokić-Begić, N., & Sangster Jokić, C. (2022)	To explore the nocebo effect focuses on (1) the mechanisms underlying the nocebo effect, (2) the characteristics of participants exhibiting a more intensive nocebo effect, and (3) the circumstances that might reduce or prevent the nocebo effect.	N = 2614	Several factors related to the conditioning procedure, expectations, and personality traits of the participants are associated with the nocebo effect, providing valuable insights into the potential mechanisms underlying this phenomenon.	■ Prior Expectations and Learning ■ Socio-demographic characteristics ■ Personality and individual differences
37.	Blease et al. (2023)	To describe potential ways in which open notes may cause nocebo effects in patients.	Not Applicable (NA)	The implementation of 'closed notes' requires some suggestions on how health systems and physicians can adapt to this innovation to mitigate the potential risk of nocebo effects that may result from this new approach.	■ Communication of information and patient-physician relationship ■ Setting
38.	Neumann et al. (2022)	To provide a comprehensive summary of the significance of placebo and nocebo effects in the fields of medicine and nutrition research.	N = 37	By improving their understanding of contextual factors, the scientific community can enhance the precision of their measurement and analysis of the effects of diet modifications in primary studies. Additionally, it is suggested that systematic medical research incorporate experimental manipulation of placebo and nocebo effects to assess their influence on patient outcomes.	■ Prior Expectations and Learning ■ Personality and individual differences ■ Communication of information and patient-physician relationship ■ Setting
39.	Zhang et al. (2022)	To assess the distribution and possible predictors of nocebo effects in primary headache treatments.	N = 31 2020	The nocebo effect was found to have a significant positive association with longer treatment duration, a high percentage of participants who received active medication, a multicenter research design, high body mass index, being female, prior treatment experiences, and a large proportion of patients with migraine headaches accompanied by an aura.	■ Prior Expectations and Learning ■ Socio-demographic characteristics ■ Personality and individual differences
40.	T. Watanabe et al. (2023)	To investigate the incidence of nocebo responses in analgesic trials of third molar removal in dentistry, to better understand the potential impact of negative expectations on treatment outcomes and inform clinical practice.	N = 8468	Patients who received a placebo reported adverse events (AEs) at a similar rate to those who received active treatment. This suggests that many AEs associated with postoperative analgesic medication may be attributed to the nocebo effect.	■ Prior Expectations and Learning ■ Drug characteristics
41.	Rooney et al. (2023)	To conduct a thorough examination and meta-analysis of existing research on the nocebo phenomenon, aiming to measure its magnitude across various outcomes and explore the factors that influence it.	N = 8219	The nocebo effect showed varying impacts across somatic outcomes and emotional states, presenting a moderate effect size overall. Moreover, it revealed a consistent inducibility across various somatic health conditions.	■ Prior Expectations and Learning ■ Personality and individual differences ■ Communication of information and patient-physician relationship
42.	E. Frisaldi et al. (2023)	To summarize the current understanding of placebo and nocebo effects linked to pharmacological interventions, along with an exploration of their underlying mechanisms.	N = 158 312	The characterization of mechanisms associated with placebo and/or nocebo effects in various medical conditions was demonstrated. These include pain, neurological disorders, mental health conditions, immune and endocrine system responses, cardiovascular and respiratory functions, gastrointestinal issues, and more.	■ Drug characteristics ■ Neurodegenerative conditions ■ Inflammatory conditions
43.	Frisaldi et al. (2024)	To characterize the placebo and nocebo responses observed in placebo-controlled randomized clinical trials (RCTs) focusing on painful diabetic neuropathy (PDN).	N = 2425	The importance of contextual elements, including confidence in PDN treatments, patients past adverse encounters, the duration of the intervention, and the information disclosed to patients before their participation confirm the magnitude of placebo and nocebo effects.	■ Prior Expectations and Learning ■ Setting ■ Neurodegenerative conditions

Legend:  = Missing data, the absence of expected sample size information.

Not Applicable (NA) = when sample size information is irrelevant or not provided in the source material.

### 3.2 Risk of bias across reviews

The quality assessment revealed that none of the 43 reviews had received a poor-quality rating. All the reviews and meta-analyses were based on a focused question that was adequately formulated and described. Of these, seven reviews adequately fulfilled all eight criteria of the checklist, including a systematic literature search strategy, an independent screening of full-text articles, and an assessment of publication bias. Nine of the systematic reviews assessed publication bias, whereas the remaining thirteen either did not report it (five) or it was not possible to apply and/or report these criteria (eight). Ten of the included meta-analyses assessed the degree of heterogeneity. Instead, one of the main limitations related to the quality assessment of reviews was that not all categories could be applied to the different types of literature reviews, based on their respective objectives and methodologies. The risk of bias for each study included in the review is shown in the Supplementary Material.

### 3.3 Synthesis of results

Through the thematic analysis conducted across the 43 reviews, we have identified nine categories of risk factors predicting the nocebo effect. Results are summarized in Table 2.

1. **Prior expectations and learning.** Negative expectations and learning are among the most relevant risk factors associated with the nocebo effect. Expectations can be engendered by different factors, including direct information, suggestions, and social cues (Wager and Atlas, 2015). Learning in its classical meaning (i.e., conditioning) occurs when a person who has a previous exposure to a stimulus reacts to it, and then responds to the same stimulus through associative processes in a similar manner. However, expectancy and learning are not mutually exclusive (Colagiuri et al., 2015). Cognitive theories of conditioning postulate that this process can also be mediated by expectations, whereby previous experiences may build up to the point of shaping patients' expectations about the course of their illness (Dodd et al., 2017). In their review, Meeuwis and colleagues (Meeuwis et al., 2021) underlined the role of conscious expectations in nocebo effects on both open-label and closed-label verbal suggestions for the itch. They highlighted that open-label verbal suggestions influenced conscious expectations, while closed-label (concealed) suggestions directly impacted itch levels without involving conscious expectations, possibly hinting at a role of learning. Social factors can also drive expectations, as seeing someone report side effects after receiving medical treatment can increase the likelihood of a similar nocebo effect (Petrie and Rief, 2019). Meanwhile, previous positive experiences may enhance placebo analgesic effects, while negative experiences can induce nocebo effects (Colloca and Benedetti, 2016). This category receives additional validation from a recent umbrella review conducted by Frisaldi et al. (2023). Their extensive analysis has demonstrated that, in the context of nocebo effects, the manipulation of expectations, conditioning, or a combination of both has proven effective in eliciting nocebo responses across diverse domains, such as pain perception, skin dryness, nausea, and cognitive performance (Frisaldi et al., 2023).
2. **Socio-demographic characteristics.** A recurrent result among the considered reviews is that women are more likely to experience nocebo effects across a range of medical conditions (Kravvariti et al., 2018). However, the explanation for any gender difference is likely complex and multifactorial. For example, in a narrative review by Manaï et al. (2019) on how to prevent, minimize, or extinguish nocebo effects in pain, women were more affected by conditioning than by verbal suggestions, whereas men responded strongly to verbal suggestions. However, it is hard to rule out the role of confounders such as anxiety, which is associated with higher nocebo effects and is more prevalent in females than in males (Vambheim and Flaten, 2017). Future research is needed to gain knowledge on whether the interaction between gender and cognition may influence nocebo effects in experimental or clinical settings (Data-Franco and Berk, 2013). Beyond gender, also older age, lower levels of socioeconomic status, as well as living in rural regions, are all factors that have been associated with adverse symptoms related to the nocebo effect, such as headache, back, and joint pain, intolerance to food, and sexual dysfunction (Data-Franco and Berk, 2013). Instead, mixed findings have been found regarding the level of education. As reported (Bavbek et al., 2015), graduation from college compared to high school or primary school may be associated with increased expectations or information-generated effects, since well-educated subjects will be more likely open to receiving information from different sources and may thus be more at risk of developing negative expectations.
3. **Personality and individual differences.** Some studies have addressed the role of personality as a predictive factor of nocebo effects and adverse event reporting. Traits such as neuroticism, pessimism, and type A personality may increase the risks for such phenomena (Data-Franco and Berk, 2013). As reported by a systematic review conducted by Kern (2020) neuroticism is the most often reported personality trait with a positive correlation with the nocebo effect. Moreover, pessimism is relatively consistently associated with nocebo, with an equally positive correlation between low optimism and the tendency to perceive negative effects (Bagarić et al., 2021). All these characteristics are more frequently found in the type A personality (aggressive/competitive/hostile personalities), which appears to be three times more likely than behavior pattern B to be associated with a nocebo effect. Fear and anxiety are also positively associated with the nocebo effect and have been found to increase the probability of negative effects of treatment (Planès et al., 2016a, Planès et al., 2016b). A review of contemporary experimental research pointed out that highly anxious people are prone to heightened attention to their body and bodily sensations and could therefore be more susceptible to the nocebo effect (Manaï et al., 2019). Other studies also confirm that patients with conditions such as depression or anxiety have a higher tendency towards somatization, which has been shown to result in increased reports of side effects. Another characteristic that might be relevant to the nocebo effect is anxiety sensitivity, namely, a fear of anxiety itself because of the belief that anxiety can have detrimental physical, mental, and social consequences. Among anxiety-sensitive individuals, the expectation of unpleasant symptoms and a consequent increase in anxiety might further heighten fear and related bodily sensations, thus leading to stronger nocebo effects. While some preliminary findings have suggested that anxiety sensitivity is associated with the nocebo effect, further research examining this topic is required (Bagarić et al., 2021). Other traits that seem to positively correlate with the nocebo effects are suggestibility and pain catastrophizing. Suggestibility, especially in terms of trait-like characteristics facilitating body sensations (e.g., physical suggestibility), has been linked to nocebo effects. Catastrophizing, an important psychological factor for pain management therapies is also found to be relevant for nocebo (Blasini et al., 2017; Meeuwis et al., 2021). The nocebo effect is significantly more pronounced in clinical populations than in healthy ones. Multiple factors, such as higher baseline anxiety, may predispose clinical populations to experience greater nocebo effects. Combining these findings, nocebo effects seem to consistently manifest across various somatic outcomes, such as pain, nausea, and headache (Rooney et al., 2023).
4. **Neurodegenerative conditions.** Some neurological modifications may also increase susceptibility to nocebo reactions (Benedetti et al., 2016; Skyt et al., 2020). In the field of brain diseases, the highest nocebo dropout rate has been observed in Parkinson's disease (PD) (Stathis et al., 2013). Human experimental evidence suggests that negative expectations could result in motor deterioration in patients with PD (Frisaldi et al., 2024). PET studies showed that high placebo effects were associated with greater dopamine (DA) and opioid activity in the nucleus accumbens, whereas nocebo effects were associated with a deactivation of DA and opioid release (Frisaldi et al., 2015). Both systems modulate several processes, including the regulation of reward and affective states. Thus, increased nocebo should be expected in PD, although DA replacement therapy results in changes in many aspects of neural activity within the entire basal ganglia cortical networks that are not yet fully understood. Not considering the nocebo dropout rate during short-term interventions for headache and multiple sclerosis (MS), the lowest nocebo dropout rate has been observed in restless legs syndrome (RLS) (Silva et al., 2017) and during disease-modifying therapies (DMTs) in MS (Papadopoulos and Mitsikostas, 2010). A meta-analysis conducted by Leal Rato (Leal Rato et al., 2019) and colleagues that included 236 randomized control trials identified that the magnitude of the nocebo effect in Parkinson's disease (PD) is substantial. The results demonstrated that most placebo-treated PD patients suffered adverse event symptoms (56%), providing evidence of a strong negative effect of an inert intervention compared to other neurological diseases. Overall, it is improbable that the nocebo effect is restricted to a particular disease or a singular pathophysiological process, but it seems that patient populations, such as those with PD, may be more susceptible to it. While the literature has shown the relevance of the nocebo effect in Parkinson's disease, a common belief among neurologists is that the nocebo effect may also be relevant in other neurological conditions, such as multiple sclerosis or epilepsy (Spanou et al., 2019).
5. **Inflammatory conditions.** In the context of inflammatory diseases, neurogenic inflammation assumes a pivotal role in pain hypersensitivity and locally exaggerated immune reactions, although its susceptibility to conditioning remains insufficiently explored (D'Amico et al., 2021a, D'Amico et al., 2021b; Linsenbardt et al., 2015). Immune-related mechanisms may underpin the intricate interplay between neurogenic inflammation and locally exaggerated immune responses (Meeuwis et al., 2021). Nocebo effects, particularly in the context of inflammatory conditions, involve intricate interactions with the immune system. Several immune-related mechanisms contribute to the manifestation of nocebo responses. This includes the release of pro-inflammatory cytokines, the bidirectional communication between the nervous and immune systems (neuroimmune interactions), and the field of psychoneuroimmunology, which explores the connections between psychological processes and immune responses (Frisaldi et al., 2015, 2023). The release of inflammatory mediators and the neurobiology-associated nocebo effects also play a role (Zhang et al., 2022). A noteworthy nocebo effect in this field is an unexplained, unfavorable therapeutic response following a switch to biosimilars, often followed by a beneficial effect upon reverting to the innovator drug (Kristensen et al., 2018). Notably, the biosimilar retention rate at the third infusion demonstrated substantial efficacy, reaching 85% among patients with inflammatory bowel disease (IBD), rheumatoid arthritis (RA), or axial spondyloarthritis (AS) (Pouillon et al., 2018). Within the spectrum of neurological conditions, the nocebo effect exhibits significant variation. Specifically, in chronic inflammatory demyelinating polyneuropathy (CIDP), the nocebo effect is notably smaller compared to other neurological diseases, as reported in a comprehensive systematic review and meta-analysis (Zis et al., 2020). One plausible explanation lies in the fact that CIDP primarily affects the peripheral nervous system, distinguishing it from other disorders that predominantly impact the central nervous system. Comorbidity with somatoform disorders and alterations in dopamine pathways within the brain have been suggested as potential contributors to this observed difference. The impact of the route of administration on the nocebo effect remains a subject of debate. In various analyses, the route of administration played a role in nocebo dropout rates. For instance, trials involving botulin toxin for the prophylactic treatment of primary headaches exhibited a significantly lower nocebo dropout rate compared to oral medication (Zhang et al., 2022). However, in multiple sclerosis, the route of administration did not significantly affect nocebo rates. In a meta-analysis, subcutaneous delivery of immunoglobulin primarily caused adverse events related to the injection site, yet the nocebo dropout rates did not show dependency on the route of drug administration (Ma et al., 2019a, Ma et al., 2019b). In the exploration of nocebo responses within the experimental endotoxemia model, individuals prone to nocebo effects reported significantly more bodily sickness symptoms. This observation suggests a link between the perception of symptoms and the influence on perceived treatment allocation (Benson and Elsenbruch, 2019). Overall mild, benign ailments commonly reported by healthy individuals may be misattributed as unwanted drug effects in pharmacological trials. In the realm of inflammatory bowel disease trials, nocebo responses take on a distinct character, manifesting as an increased reporting of adverse events when patients transition from an established, albeit expensive, biologic therapy to a more cost-effective approach with biosimilars (D'Amico et al., 2021a, D'Amico et al., 2021b; Zis and Mitsikostas, 2018). Furthermore, the specific medications under investigation, particularly those targeting the immune system, may influence nocebo responses (Frisaldi et al., 2023).
6. **Communication of information and patient-physician relationship.** Preliminary evidence indicates that communication and education techniques might be effective in reducing nocebo effects induced through instruction (Rooney et al., 2023). Non-verbal communication, such as eye contact, posture, grimace, and movement style during the encounter with a patient is important, as these forms of communication can predispose patients to experience nocebo effects both consciously and subconsciously (Kravvariti et al., 2018). Compared to a cold communication style (i.e., directing gaze and body posture away from participants and no empathic remarks), a warm communication style (i.e., gazing at the patient, welcoming in a friendly manner, an open body posture, and adding empathic remarks) of clinicians resulted in positive expectations (e.g., expectations of shorter pain duration), decrease in anxiety and negative mood (Daniali and Flaten, 2019). Overall, a cold communication style resulted in higher anxiety levels and expectations of longer pain duration in patients. In this regard, the review of Hansen and Zech (2019) emphasizes the importance that medical personnel adopt explicit/implicit positive expressions towards their patients, otherwise, this could lead to reduced effectiveness of the treatment through nocebo-like mechanisms. Indeed, verbal, and nonverbal communications between physicians and nursing staff contain numerous unintentional negative suggestions that may trigger a nocebo effect: body posture, tone of voice, a shrug of shoulders, frown, or furrowed brow (Planès et al., 2016a, Planès et al., 2016b). Attitude as another component of non-verbal communication also plays a key role in predicting the occurrence of a nocebo effect: physicians who are more encouraging, kind, affectionate, and provide a clear diagnosis appear to be more effective, for example, in reducing levels of perceived pain and the time needed to improve than physicians who adopt a more rigid attitude and offered no consolation (Data-Franco and Berk, 2013; Planès et al., 2016a, Planès et al., 2016b). Furthermore, informed consent to therapeutic interventions is part of the encounter in which negative anticipation is frequently introduced. Potential adverse events, although rare, frequently monopolize discussions and tend to be framed negatively; for example, physicians will usually state the small percentages of patients who experience adverse events, rather than the large percentage of patients who tolerate the medication well (Planès et al., 2016a, Planès et al., 2016b). Negatively framed information is also associated with higher expectations of side effects, whereas framing and customization of information help to develop more functional treatment expectations and prevent nocebo effects induced by expectation (Smith et al., 2020). However, the physician's manner during the discussion might be a more pertinent risk factor for nocebo effects than the actual content of the information provided, as well as the interactions with nonmedical staff and fellow patients (Evers et al., 2018, Evers et al., 2018b). However, it is important to keep in mind that anxious or pessimistic patients can also actively find negative information by themselves (pairs, the Internet, and leaflets on drugs). Many sources of medical information on the Internet and conventional media overstate the negative effects of treatments and patients seeking consultation in online forums and blogs might be susceptible to the nocebo effect due to the misguided beliefs stemming from this overly negative information. This, in turn, can lead to drug intolerance and non-adherence to medications (Evers et al., 2018, Evers et al., 2018b; Planès et al., 2016a, Planès et al., 2016b). Social features such as the physician's reputation and references, attire, grooming, beliefs, and manners can also affect the patient's expectations of the treatment outcome. A qualitative systematic review (Daniali and Flaten, 2019)revealed that even experimenters' and/or clinicians' status may determine the nocebo effect: for example, higher professional status and higher confidence of experimenters/clinicians led to lower pain reports, more accurate pain ratings, and better physical and emotional states. The nature of the therapeutic alliance may also be a driver of the nocebo effect, with a hostile-dependent relationship being an exemplar (Dodd et al., 2017). Overall, several factors converge in the construction of an authentic interpersonal relationship that can buffer against nocebo effects: patient-oriented information, an empathic attitude on the part of the therapist that inspires trust, as well as empowerment to support self-efficacy and individual responsibility (Neumann et al., 2022; Smith et al., 2020).
7. **Drug characteristics.** Not much research has investigated whether the type of medication received by the patient can significantly contribute to the nocebo effect. The available evidence seems to suggest that additional marketing features of a drug, such as price and labeling, are important factors that can influence the therapeutic effects (Planès et al., 2016a, Planès et al., 2016b). Patients and physicians generally consider generic drugs to have lower efficacy and be associated with more adverse effects than their brand-name counterparts. Hence, the use of general labeling has been associated with medication non-adherence. Furthermore, brand labeling of the medication can increase placebo effects whereas generic labeling of the medication is associated with higher rates of nocebo effects (Faasse et al., 2013). Nocebo effects are also most prevalent in the initial period of trying a medication that is new to the patient, and the fear of experiencing adverse events associated with generic medication might be rooted in the fact that these medicines are often newer to the market and physicians have less experience in using them than branded medication. However, as pointed out by several reviews (Petrie and Rief, 2019; Spanou et al., 2019), if the tablets had a generic label, the placebo tablets were less effective compared to active ibuprofen. Fewer side effects were attributed to placebo tablets with brand-name labeling compared to placebo tablets with a generic label. The nocebo effect can also be created by more subtle branding cues when patients are switched from a branded to a generic medicine: drug switches from branded to generic can result in increased reports of side effects and complaints that the new drug is less effective (Petrie and Rief, 2019). Also, certain features of medications that are unrelated to their main pharmacological action, such as the color, odor, or route of administration, can influence therapeutic efficacy. Finally, injectable therapies induce stronger placebo effects and have lower rates of nocebo effects than oral medications as proven by studies of therapies for migraine or osteoarthritis pain (Kravvariti et al., 2018).
8. **Setting.** Besides patients and physicians, other features of the healthcare setting might introduce positive or negative anticipation of treatment effects. Examples include physical properties of the medical setting such as the type or quality of lighting, sound, architecture, interior design, and technology, as well as the ease and affordability of access to care. Kravvariti et al. (2018) highlighted the clinical relevance of contextual factors as triggers of nocebo effects in the healthcare setting, in terms of environment, architecture, and interior design, which should not be overlooked. The use of facilities where evidence-based design such as furnishing, colors, artwork, light, outside views, temperature, soothing sound, and music are adopted, positively impacts patients' outcomes thanks to the creation of a proper healing setting, which can reduce nocebo-induced adverse symptoms These contextual factors act as a continuous outcome-relevant influence throughout the entire process, that is, during anamnesis, diagnosis, implementation advice, and the final evaluation (Neumann et al., 2022).
9. **Self-awareness.** Some evidence suggests that the level of awareness regarding certain stimuli may influence outcomes. Indeed, there is a large literature suggesting that behavior can be motivated by stimuli that are not consciously perceived because they are presented at low intensities or masked from conscious awareness (Custers and Aarts, 2010)sometimes referred to as subliminal stimuli (Powers, 1973). However, the role of awareness not only for placebo but also for nocebo effects is still a matter of debate. In their comprehensive systematic review, Webster and colleagues (Webster et al., 2016) claim that there is little evidence that self-awareness increases the likelihood of a nocebo effect. Both placebo and nocebo effects can be triggered by non-conscious cues (i.e., operating outside of conscious awareness), mixing these results with the ones from conditioning mechanisms (Jensen et al., 2012). For example, Colloca and Benedetti (2016) in their review pointed out that patients undergoing pain treatment respond more positively when they are aware of receiving pain medication. Brain imaging studies have recently extended and corroborated these results by demonstrating that being aware of receiving a treatment potentiates the pharmacological analgesic effect of remifentanil in healthy subjects receiving acute thermal painful stimulation (Colloca and Benedetti, 2016). The focus on monitoring the side effects of one's own body and the consequent distraction from concentrating on the expected result of the drug leads to an increase in symptoms and the absence of a placebo effect (Hansen and Zech, 2019).

**Table 2:** Nocebo risk factors.

Risk Factor(s)	Synthesis
**1. Prior Expectations and Learning**	■ Expectations generated as the product of cognitive engagement involve the subjectively experienced likelihood of a future effect, often induced by verbal suggestions, which promotes the nocebo effect. ■ Learning mechanisms can involve classical conditioning, a process whereby the repeated association of an unconditioned effect with a conditioned stimulus increases the likelihood of the nocebo effect. ■ Negative beliefs about the overuse of medications are associated with increased side-effect expectations, and increased perceived sensitivity to medicines is associated with increased side-effect expectations.
**2. Socio-demographic characteristics**	■ Women are more affected by conditioning than by verbal suggestions, while men respond stronger to verbal suggestions. ■ Old age, lower levels of education and socioeconomic status, and living in rural regions are all positively related to nocebo reactions.
**3. Personality and individual differences**	■ Anxiety, depression, a tendency toward somatization, and symptom amplification positively correlate with the nocebo effect. ■ Type A personalities, characterized by being aggressive, competitive, hostile, and pessimistic, are more likely to experience adverse symptoms. ■ The personality trait most frequently associated with the nocebo effect is neuroticism.
**4. Neurodegenerative conditions**	■ Clinical problems, such as dementia, psychosis, hallucinations, orthostatic hypotension, and sleep disorders, result in a substantial nocebo effect. ■ Patients with mild cognitive impairment or dementia related to Alzheimer's disease, a loss of prefrontal functional connectivity, and executive control, are associated with a higher nocebo effect. ■ Considering the pronounced neuronal degeneration in the dorsolateral prefrontal cortex, orbitofrontal cortex, and anterior cingulate cortex in Alzheimer's disease, it is reasonable to anticipate a disruption of placebo responsiveness in these patients.
**5. Inflammatory conditions**	■ Integrate findings with existing literature on placebo/nocebo effects, emphasizing the interconnectedness of psychological, neurological, and immunological factors within the framework of biosimilar challenges in inflammatory settings. ■ The influence of patient-related factors, including comorbidities, changes in dopamine pathways, and somatoform disorders, on the manifestation of the nocebo effect. ■ Investigate instances of the nocebo effect in patients transitioning to biosimilars, uncovering unfavorable therapeutic responses and potential benefits upon returning to the innovator drug.
**6. Communication of information and patient-physician relationship**	■ Inadequate non-verbal behaviors, such as lack of eye contact or body gestures, are associated with an increased perception of adverse symptoms. ■ Not explaining the nocebo effects and focusing on losses rather than benefits can increase side effects. ■ A cold communication style, characterized by directing gaze and body posture away from participants, a lack of empathic remarks, and a rigid attitude, as well as a missing treatment alliance, promotes the nocebo effect.
**7. Drug characteristics**	■ Drugs with generic brand labeling are associated with an increased nocebo effect. ■ Oral medications induce a stronger nocebo effect.
**8. Setting**	■ Reduced patient trust in clinicians and perceptions of low professional status can lead to negative effects. ■ An improper healing setting, including the type or quality of lighting, sound, architecture, interior design, technology, or missing facilities, can trigger nocebo effects.
**9. Self-awareness**	■ Nocebo effects can be triggered by non-conscious cues (i.e., operating outside of conscious awareness). ■ The relation between self-awareness and the likelihood of a nocebo effect is still a matter of debate. ■ Gaps in patient awareness and understanding, may cause the perception of nocebo-related adverse symptoms.

### 3.4 Neurobiological mechanisms

There are multiple neurobiological mechanisms implicated in the development of nocebo effects, although these are often studied without a clear connection to the identified risk and protective factors. In general, most knowledge about these mechanisms comes from the field of pain and analgesia, even though much less research has been done on nocebo effects than on placebo effects (Colloca and Benedetti, 2016; Planès et al., 2016a, Planès et al., 2016b). The following endogenous substances have been identified so far in this setting: cholecystokinin, dopamine, corticoids, and opioids. The mixed cholecystokinin (CCK) type A/B receptor antagonist, proglumide blocks nocebo hyperalgesia with no effect on cortisol and adrenocorticotropic hormone supporting the direct role of CCK in the hyperalgesic nocebo effect. Multiple hypotheses have been put forward explaining the nocebo effect including endogenous substances and psychosocial mediators (Manchikanti et al., 2011). Overall, it appears that there is an interaction and a link between cholecystokinin (CCK), pain, and anxiety. This may help explain the mechanisms underlying how certain individual differences, particularly anxiety, may facilitate the nocebo effect. Benedetti et al. (2005) found that expectation-induced hyperalgesia can be blocked by administering proglumide, a CCK-receptor antagonist. Recent studies (Frisaldi et al., 2015) have shown that nocebo pain effects induced in post-operative patients by negative expectations regarding a saline infusion could be prevented by the CCK antagonist proglumide, a nonspecific CCK-1, and CCK-2.

The role of certain neurodegenerative diseases as risk factors for nocebo effects may be linked to dopamine. High placebo effects are associated with greater dopaminergic and opioid activity in the nucleus accumbens (significant decrease of the l-receptors’ binding potential), whereas nocebo effects are associated with a deactivation of dopamine (Skyt et al., 2020). Another study about the role of corticoids (Benedetti et al., 2006) showed that high placebo effects are associated with greater dopaminergic and opioid activity in the nucleus accumbens (significant decrease of the l-receptors’ binding potential), whereas nocebo effects are associated with a deactivation of dopamine.

Patient-physician relational aspects, as well as setting components, may exert their influence through certain biochemical mediators. A study about the role of corticoids (Planès et al., 2016a, Planès et al., 2016b) showed that verbally induced nocebo hyperalgesia was associated with hyperactivity of the hypothalamic-pituitary-adrenal (HPA) axis, as assessed through adrenocorticotropic hormone and cortisol plasma concentrations. The blocking of the nocebo effect is not mediated by endogenous opiates since the infusion of naloxone does not prevent the effects of proglumide (Planès et al., 2016a, Planès et al., 2016b). The opioidergic and the CCKergic systems may be activated by opposite expectations of either analgesia or hyperalgesia, respectively. Verbal suggestions of a positive outcome (pain decrease) activate endogenous l-opioid neurotransmission, while suggestions of a negative outcome (pain increase) activate CCK-A and/or CCK-B receptors (Planès et al., 2016a, Planès et al., 2016b). The opioid antagonist naloxone does not prevent the attenuating effect of proglumide on the nocebo effect. Although nocebo effects are often described as the negative counterpart of the placebo effect with opposite effects on pain, these findings suggest that placebo and nocebo effects do not involve opposite neurotransmission activity, at least not in the endogenous opioid system (Planès et al., 2016a, Planès et al., 2016b; Scott et al., 2008).

Neuroimaging techniques have also highlighted important contributions to our knowledge of nocebo hyperalgesia, especially when related to expectations. Inducing negative expectations results in both amplified unpleasantness of innocuous thermal stimuli as assessed by psychophysical pain measures (verbal subject report) and increased fMRI effects in the anterior cingulate cortex and a region including the parietal operculum and posterior insula. Together with the hippocampus and the prefrontal cortex, these are regions also involved in pain anticipation. Changes in the hypothalamic-pituitary-adrenal axis, including rises in adrenocorticotrophic hormone and cortisol, have been linked to pain perception and expectation. Neuroimaging studies have examined this phenomenon: a positron emission tomography study reported changes in μ-opioid and dopamine D2/D3 neurotransmission with the nocebo effect, and functional magnetic resonance imaging studies have suggested the involvement of specific brain structures, such as the anterior cingulate, insula, and the prefrontal cortex (Frisaldi et al., 2015). Due to the limited number of studies, more research is needed to draw firm conclusions.

## 4 Discussion

The present review of reviews offers a synthesized overview of the main risk factors associated with the nocebo effect, considering its impact and pervasiveness in the clinical context (Colloca and Miller, 2011). These factors span from individual-independent characteristics, such as contextual cues and physical properties of the treatment, to psychological and personal characteristics such as expectations, affective states, and personality traits. Crucial risk factors for the nocebo effect are prior expectations and learning, which often act synergistically towards its development and maintenance (Meeuwis et al., 2021). Instead, the role played by awareness is still a topic of open debate on which future studies should focus. Patients, as catalysts of the effect, often take an active role in shaping nocebo effects (Colloca and Miller, 2011). Moreover, findings on the impact of negative communication styles and attitudes suggest how a patient-centered approach that is rooted in demonstrating care and empathy can positively enhance a patient's experience within the clinical environment and activate psycho-sociobiological adaptations that can counteract the nocebo phenomenon (Evers, 2017; Blasini et al., 2018). Socio-demographic factors, such as gender and level of education, also require further studies that can confirm whether they are risk factors and to what extent. For example, a lower level of education could more easily lead to misconceptions and thus represent a potential risk factor (Bizzi et al., 2019). The nocebo effect has occasionally been referred to as the 'evil twin' of the placebo effect. If this were true, one would expect the risk factors for a nocebo effect to be the inverse of the predictors for a placebo effect (Glick, 2016). As Webster and colleagues have already pointed out in their systematic review (2016), the mechanisms advocated in our review appear to be like those previously identified for placebo effects. The results of our review can therefore be compared with those of Webster's analysis, which clustered the basic risk factors in six different categories (demographics; clinical characteristics; expectations; anxiety; personality; miscellaneous). Specifically, of these six categories, three were retained and confirmed in our study (socio-demographics, personality, and expectations). The category ‘anxiety’ as well as ‘clinical characteristic’, in our case, was merged into the category ‘personality and individual differences’, in which other characteristics (such as pessimism, anxiety, and catastrophizing) were included. The main difference between the two works is that in the present review, the category defined by Webster as ‘miscellaneous’ has been separated into seven new categories (neurodegenerative conditions; inflammatory conditions; communication of information and patient-physician relationship; drug characteristics; setting; and self-awareness). Furthermore, it should be emphasized that the nine identified categories are not to be conceived as rigidly separated from one another but instead characterized by ‘permeable’ boundaries: factors across categories may be interdependent and feed off each other to induce a nocebo effect. For instance, patients receiving intravenous therapy as a group in the same infusion suite can share stories, experiences, and opinions, which might influence individual perceptions and trigger nocebo effects. At the same time, personality and individual differences can also determine how patients interpret the information that they receive. The discussion on psychoneuroimmunology (PNI) is crucial in unraveling the complexities of the nocebo effect. PNI, which delves into the interplay among psychological, neurological, and immunological factors, plays a pivotal role in understanding how behavior and immunity reciprocally influence each other (Reza et al., 2023). This field challenges the conventional view of the immune system as an autonomous entity and offers a comprehensive bio-psycho-social perspective on health and illness. By examining the dynamic interactions among the nervous, endocrine, and immune systems, PNI contributes significantly to our comprehension of the interplay between psychosocial variables, health, and illness (Zachariae, 2009). In this context, the present review emphasizes various factors influencing the nocebo effect, with particular attention to the impact of prior adversity and its role in vulnerability. While our primary focus has centered on psychological and contextual factors, it is imperative to acknowledge the broader ecological perspective presented in the work of Harvey (2023). The evolutionary viewpoint on threat-responsive neuroinflammation provides valuable insights into the physiological dimensions of the nocebo effect. Harvey's investigation of shared neuroimmune pathways in pain, somatization, anxiety, and PTSD contributes to understanding cross-domain sensitization in the nocebo phenomenon (Harvey, 2023). The integration of ecological perspectives enriches our understanding of the multifaceted nature of the nocebo effect, offering a broader context for personalized recovery approaches that encompass physical, mental, and social aspects (Rossettini et al., n.d.; 2018). This integrative approach aims to shed light on the therapeutic prospects emerging from these phenomena within the conceptual foundations of PNI-based mind-body therapies.

This review is not immune from some limitations. First, there is considerable heterogeneity in both the studies and the contexts in which the nocebo effect is examined. This variation is primarily observed in healthcare settings dealing with neurodegenerative or inflammatory diseases. It also extends to the general population, where individuals are exposed to inert substances to assess various baseline or experimental factors. Secondly, we noticed how the mixed findings regarding the category 'self-awareness' make it necessary to carry out further studies to claim that this factor can predispose to the nocebo effect. Overall, given the increasing number of studies, subsequent meta-analyses are needed to aggregate in a more analytical and precise way those data useful to understand how each specific risk factor may have different effects. Another limitation arises from the inclusion of solely English-language studies, introducing the possibility of a publication bias. This bias may also originate from the tendency of published results included in reviews to predominantly report significant effects. However, placebo and nocebo effects can be viewed as significant or non-significant results, based on the specific research question. Moreover, the inclusion of grey literature is meant to mitigate this phenomenon.

## 5 . Conclusions and future directions

The current review of reviews provides a comprehensive and up-to-date summary of the risk factors that predispose to or contribute to the nocebo effect. It is presented here as the most exhaustive synthesis available in the literature, providing a critical starting point for further investigation into the counterpart of the placebo effect. The findings highlight some of the risk factors underlying the nocebo effect and its potential impact on patient outcomes. Delving into the connections among the psyche, neural, and endocrine functions, as well as immune responses, psychoneuroimmunoendocrinology focuses on applying this knowledge to medical treatment across various conditions (González-Díaz et al., 2017). These include immune disorders, autoimmune diseases, neoplastic conditions, and endocrine disorders. Psychoneuroendocrinology is a field of study that explores the intricate connections between psychological processes, the nervous system, and the endocrine (hormonal) system (Barbiani and Benedetti, 2020). It examines how psychological factors, such as stress and emotions, can influence the endocrine system and, in turn, impact various physiological functions and health outcomes. In the context of nocebo effects, psychoneuroendocrinology investigates how psychological factors, such as expectations, beliefs, and emotions, can activate the endocrine system to induce adverse reactions or symptoms. This field examines the interplay between psychological states and hormonal responses that contribute to the manifestation of nocebo effects, wherein the anticipation of negative outcomes leads to the actual experience of adverse symptoms or side effects. Notably, the clinical implications of psychoneuroimmunoendocrinology are particularly pronounced in the context of nocebo effects (Colloca et al., 2019). Epigenetic factors and significant stressors, operating through diverse pathways and neurotransmitters, play a pivotal role in modulating the psychoneuroimmunoendocrine axis, contributing to the onset of disease (González-Díaz et al., 2017). This reinterpretation emphasizes the relevance of psychoneuroimmunoendocrinology, highlighting its crucial role in understanding and addressing clinical implications, especially concerning the manifestation of nocebo effects across various pathologies.

Addressing the gap between clinical research, focused on minimizing or eradicating placebo mechanisms, and clinical practice, requiring an understanding of an intervention's maximum potential, is crucial (Petrie and Rief, 2019). The exploration into identifying individuals prone to nocebo effects at the commencement of treatment underlines the need for further empirical evidence through future studies. Patient expectations serve as a valuable starting point for integrating these factors into clinical practice. Educating patients about their expectations has shown promise in improving outcomes, as seen in reduced disability outcomes after cardiac surgery and decreased postoperative pain through preoperative education about coping strategies (Colloca and Barsky, 2020). These implications extend to research design in clinical trials, emphasizing the necessity for no-intervention groups, standardized information presentation, and cautious interpretation of meta-analyses lacking uniformity (Colloca and Finniss, 2012). This comprehensive understanding of placebo and nocebo effects informs a nuanced approach to treatment, incorporating patient expectations and communication strategies for enhanced clinical outcomes (Colloca and Barsky, 2020).

The implication of this approach is that to be truly patient-centered, medicine must pay attention to the predictive process underlying the perception of symptoms, and thus assess which efficient courses of action can lead the brain to predict the health of the organism (Ongaro and Kaptchuk, 2018). Several strategies can reduce or even prevent the nocebo effect, including a reformulation of the information provided to subjects on side effects, the creation of a reassuring and protective environment when prescribing drugs, and the promotion of a trust-oriented relationship with the referring clinicians (Manaï et al., 2019). As suggested by the Bayesian brain hypothesis, what we perceive is not the world as it is, but the brain's best guess about it, continually refined by incoming sensory evidence (Friston, 2010). Applying this hypothesis to the placebo and nocebo effects, which seems a promising interpreting framework (Pagnini et al., 2023), one feels symptoms, including pain, when the hypothesis with the lowest prediction error represents an abnormal somatic event (den Bergh O et al., 2017). Furthermore, in the context of chronic pain, the brain does not merely passively perceive pain, but can also play a part in its intensification. A wide-ranging and debated issue concerns the decision of whether to provide full information to the patients about their medication and/or therapy. While this might promote more active patient engagement, informing about side effects might also cause harm (Daniali and Flaten, 2019). To manage this ethical dilemma, it is necessary to consider adopting a shared approach to reduce expectation-induced side effects while respecting patient integrity (Cohen, 2014). Factors related to the environment that might increase the adverse effects of a drug or therapy should also be considered. Overall, this study underscores the urgent need for continued research into the nocebo effect and its clinical implications, as well as the importance of improving communication and the doctor-patient relationship to better understand patients' expectations and beliefs about the adverse effects of an intervention.

## CRediT authorship contribution statement

**Francesca Grosso:** Writing – original draft, Methodology, Data curation, Conceptualization. **Diletta Barbiani:** Writing – review & editing, Supervision. **Cesare Cavalera:** Writing – review & editing, Supervision. **Eleonora Volpato:** Supervision. **Francesco Pagnini:** Writing – review & editing, Methodology, Data curation, Conceptualization.

## Declaration of competing interest

The authors declare that they have no known competing financial interests or personal relationships that could have appeared to influence the work reported in this paper.

## Data Availability

Data will be made available on request.
